# What Makes a Pattern? Matching Decoding Methods to Data in Multivariate Pattern Analysis

**DOI:** 10.3389/fnins.2012.00162

**Published:** 2012-11-23

**Authors:** Philip A. Kragel, R. McKell Carter, Scott A. Huettel

**Affiliations:** ^1^Department of Psychology and Neuroscience, Duke UniversityDurham, NC, USA; ^2^Center for Cognitive Neuroscience, Duke UniversityDurham, NC, USA; ^3^Brain Imaging and Analysis Center, Duke UniversityDurham, NC, USA

**Keywords:** fMRI, MVPA, classification, linear, non-linear

## Abstract

Research in neuroscience faces the challenge of integrating information across different spatial scales of brain function. A promising technique for harnessing information at a range of spatial scales is multivariate pattern analysis (MVPA) of functional magnetic resonance imaging (fMRI) data. While the prevalence of MVPA has increased dramatically in recent years, its typical implementations for classification of mental states utilize only a subset of the information encoded in local fMRI signals. We review published studies employing multivariate pattern classification since the technique’s introduction, which reveal an extensive focus on the improved detection power that linear classifiers provide over traditional analysis techniques. We demonstrate using simulations and a searchlight approach, however, that non-linear classifiers are capable of extracting distinct information about interactions within a local region. We conclude that for spatially localized analyses, such as searchlight and region of interest, multiple classification approaches should be compared in order to match fMRI analyses to the properties of local circuits.

## Introduction

Our ability to understand brain function is limited by the scale and accuracy with which we can quantify neural activity. Knowledge about brain function comes from different research methods, depending on the spatial scale: individual neurons (Koch, 1997; Dayan and Abbott, 2001; Gerstner and Kistler, 2002), cortical columns, larger brain regions (Rolls and Treves, 1998; Frackowiak, 2004), and functional networks spanning the whole brain (Fox et al., 2005). As one example, recording from single neurons provides exquisitely detailed temporal records of action potentials (up to the millisecond scale) but limits our coverage of the brain to a small volume surrounding the electrode a cross-section of about three square micrometers (Boulton et al., 1990). Conversely, functional magnetic resonance imaging (fMRI) provides information about neural metabolic changes, not neuronal activity, but allows an unparalleled combination of spatial resolution (down to sub-millimeter resolution) and whole brain coverage (Huettel et al., 2008). In spite of this, the spatial resolution of fMRI in typical experiments is still inadequate to describe even columnar structures (Mountcastle, 1997). Gaps between levels of description present barriers both to integrated basic science research and to a more refined treatment of mental disorders.

Recent technological, methodological, and analytical innovations have promised to bridge knowledge across levels of spatial resolution (Kim et al., 2000; Logothetis et al., 2001; Kamitani and Tong, 2005; Kriegeskorte and Bandettini, 2007; Kriegeskorte et al., 2008). Here we inspect the recent introduction of statistical learning techniques to fMRI, often grouped under the term multivariate pattern analysis (MVPA; Haynes and Rees, 2006; Norman et al., 2006; Pereira et al., 2009; Weil and Rees, 2010). MVPA simultaneously examines the disparate signals carried within a set of voxels rather than examining individual voxels in parallel, as is done in the univariate approaches that are used in the vast majority of fMRI studies. By considering multiple responses jointly, MVPA can reveal signal components that are independent of the average regional response. Extending this principle to whole brain fMRI data, MVPA can be used to provide an information-theoretic framework for the isolation of regions that uniquely represent a behavior (Hampton and O’doherty, 2007; Carter et al., 2012). In principle, MVPA also holds the capability to describe brain function at sub-voxel levels (Kriegeskorte et al., 2010), filling the glaring gap between our knowledge of small groups of neurons and the body of research describing functional characteristics of the brain.

While a number of techniques from statistical learning have been applied to fMRI data, here we focus on the use of multivariate pattern classification (MVPC) to decode mental states. Due to the poor generalization of models that utilize whole brain data, most analyses apply some form of feature selection to limit model complexity and improve generalizability. Here we utilize a common MVPC feature selection method which isolates local spheres of voxels and generates a separate model for each sphere, commonly referred to as the searchlight approach (Kriegeskorte et al., 2006). The use of searchlights focuses inference on patterning of information within a given localized area, meeting our goal of bridging voxel-wise information and regional coding.

**Figure 1:**
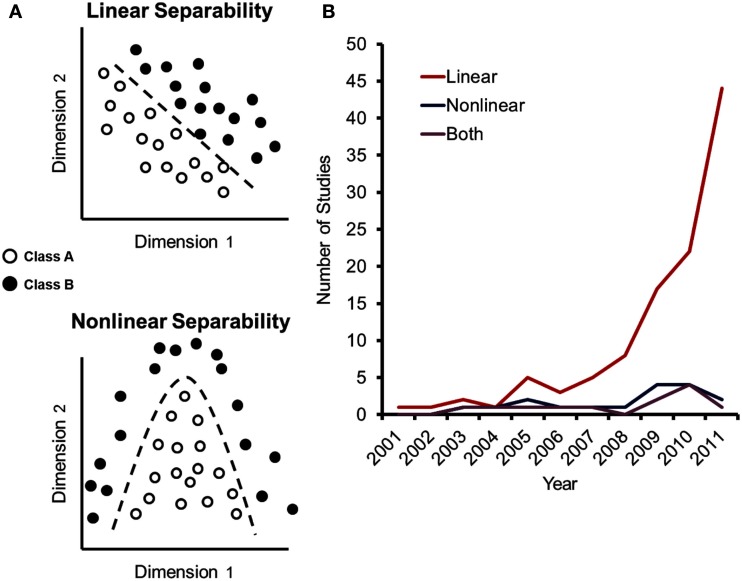
**Learning algorithms in multivariate pattern classification (MVPC) of fMRI studies**. **(A)** Pattern classification problems can be identified as linearly separable or inseparable, depending upon how component features encode information. Solutions for linear and non-linear pattern separation are depicted as detailed by Mangasarian (1965). If linearly separable, an *n*-dimensional planar surface defined by the point *x* weighted by the vector *d* and offset by the scalar γ can successfully separate the patterns *A* and *B* (*xd*−γ = 0 | *Ad – e*γ > 0, *Bd – l*γ < 0). In the case of quadratic separability shown here, an additional term can be added creating a non-linear surface that separates *A* and *B* (*xEx*’ + *xd* – γ = 0 | *A_i_EA_i_*’ + *A_i_d* – γ > 0, *B_j_EB_j_*’ + *B_j_d* – γ < 0). **(B)** Number of publications using linear and non-linear algorithms in our meta-analysis of the neuroscience literature broken down by year, showing recent growth in the use of linear rather than non-linear algorithms. The analysis was accomplished by searching PubMed on August 29, 2011 for the terms (*fMRI or MRI*) and [*MVPA or decoding or* (*pattern classification*)], identifying studies from that search that used pattern classification to study brain function – with the assistance of the AntConc corpus analysis toolkit (Anthony, 2011).

The classification algorithms used in MVPC can generally be divided into two categories. Linear classification algorithms (Figure 1A, top) use a weighted combination of signals from voxels within a feature set (e.g., a brain region) to decode perceptual or cognitive states. These methods show a measurable benefit in signal detection beyond using a univariate general linear model. However, each individual voxel must still contain information that can separate the stimuli of interest (see Mangasarian, 1965 for a mathematical description). In contrast, non-linear classification algorithms (Figure 1A, bottom) use a complex combination of information across voxels (e.g., a polynomial, sinusoid, or Gaussian function) so that even voxels that contain no useful information by themselves may still improve the classification performance of a larger set of voxels. Thus, linear and non-linear classifiers are capable of characterizing different types of neural representations (Rasmussen et al., 2011).

In addition to characterizing the type of information encoded within a brain region, comparing linear and non-linear classifiers may offer insight into how activation may be read out or manipulated through subsequent processing steps. For example, if the activation of a region can be decoded using linear classification, functionally connected regions could make use of the identified differences directly; without additional processing (for further discussion, see Norman et al., 2006; Misaki et al., 2010). Conversely, a significant non-linear classification (in the absence of linear decoding) suggests any information represented in that region will require further processing in order to be utilized (Kouh and Poggio, 2008). An example of the distinction is present in work by Kamitani and Tong (2005), where linear ensemble classifiers are sufficient to decode the orientation of perceived lines from patterns of fMRI activity in early visual cortex. Orientation information has been identified, and is being utilized immediately without extended processing. Using a non-linear classifier could allow decoding of face representations in these same voxels, even though their underlying neuronal activity would not be explicitly coding for those representations (i.e., the integration process would happen at a later stage in processing). In this regard, contrasting the decoding capability of linear and non-linear classification may give insight into whether multivariate information is at an early or late stage of a processing stream.

The capacity of non-linear classification algorithms to decode complex patterns comes with a cost. As the complexity of a classifier is increased relative to the quality and amount of data available (e.g., by increasing the number of features or by using algorithms with more parameters), the possibility of overfitting is more likely (Duda and Hart, 1973). Measures of complexity, such as the Vapnik–Chervonenkis dimension (Vapnik and Chervonenkis, 1971) or Rademacher complexity (Bartlett and Mendelson, 2001), are useful in constructing classifiers because they allow one to assess the feasibility of learning on out of sample data (Blumer et al., 1989). For example, non-linear classifiers may estimate an overly complex decision boundary, resulting in poor performance in tests of generalization. Overfitting a complex function may obscure linear relationships that are present in the data, although methods such as regularization and cross-validation have been developed to mitigate this problem (Mitchell, 1997). One such approach is the use of spatial regularization, whereby prior information about the spatial relationships of voxels is used to guide classification (Martinez-Ramon et al., 2006; Meyer and Xilin, 2008; Xiang et al., 2009). While the potential for overfitting is high in the case of fMRI (Misaki et al., 2010; Pereira and Botvinick, 2011), several studies (e.g., Hanson et al., 2004; Rasmussen et al., 2011) have demonstrated that pattern classifiers are capable of decoding information bound in non-linear relationships across multivariate samples. Thus, while overfitting is conventionally considered a drawback it can also serve as a marker for excessive model complexity.

Accordingly, the complexity of decoding algorithms should ideally reflect the way in which information is encoded. For example, the neural coding of motion in the occipital gyrus has been investigated at multiple levels of analysis. Brain structures responsible for encoding visual motion (Zeki et al., 1991; Tootell et al., 1995) have been identified using univariate approaches, while more specific information such as the direction of perceived motion has been successfully decoded using linear pattern classification (Kamitani and Tong, 2006). Indeed, the use of pattern analysis is redefining the limits of fMRI, revealing information encoded at spatial scales thought to be beyond the resolution afforded by current technology (Kamitani and Sawahata, 2010; Kriegeskorte et al., 2010), although the origin of this information is debated (Op De Beeck, 2010a,b; Shmuel et al., 2010; Freeman et al., 2011). These findings demonstrate that the improved sensitivity and use of spatial information by multivariate classifiers permit the decoding of more complex information than standard univariate approaches.

Linear and non-linear classifiers are capable of solving different types of classification problems and therefore can provide differential insight into brain function – yet, in practice they are applied to similar research questions. To characterize the usage of these two classes of decoding methods in the literature, we conducted a meta-analysis of fMRI studies employing MVPA methods. Strikingly, 110 of 115 studies (95.7%) used linear algorithms while only 16 (13.9%) utilized a non-linear approach and 11 studies implemented both methods. Although interpretability and resilience to overfitting in high dimensional datasets warrants the utilization of linear algorithms, their disproportionate use critically limits the types of information that can be decoded from patterns of neural activity. As MVPC is capable of decoding information at multiple levels of complexity and is being used at a rapidly increasing rate (Figure 1B), understanding what information is extracted by these different methods can guide future research.

## Encoding and Decoding Signals

**Figure 2:**
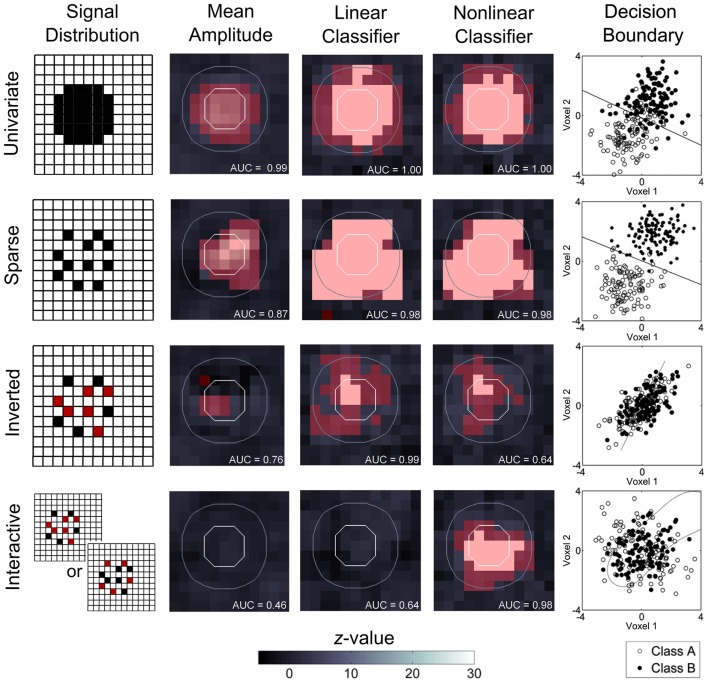
**The relative information sensitivity of different fMRI analysis approaches**. Simulated datasets comprised a 12-by-12 matrix sampled at 240 time points in which consecutive blocks of 10 time points alternate between two states (*A* and *B*). Signal discriminating the two states is present in a circle of radius 3 voxels above 10 dB white Gaussian noise. Circles marked with white lines indicate amount of encoded information, with the inner circle containing informative patterns when sampled with a searchlight, while the outer circle may contain some information as a result of spatial smoothing (Gaussian kernel with FWHM of 3 voxels). Red coloring indicates successfully decoded voxels at a family wise error corrected threshold of *p* < 0.05. Signal detection is quantified using area under the receiver operating characteristic curve (AUC). In the *univariate encoding* simulation, information that is encoded by the mean activity of each sample independently, with a homogeneous spatial distribution, is successfully decoded by all methods. For the example with *sparse encoding*, information present in the mean activity and spatial location of each sample is detected by all three analysis approaches, although MVPC provides increased sensitivity. In the *inverted encoding* simulation, detection performance is greater for both MVPC approaches than for univariate approaches. And, in the *interactive encoding* simulation, where embedded signals interact in a state dependent manner to produce information, only the non-linear approach was capable of successfully identifying the embedded signal.

To examine how a focus on linear classifiers limits the scope of MVPC, we created simulated data with different spatial distributions of information and attempted to decode them using standard univariate, linear multivariate, and non-linear multivariate models. The simulations were tailored to demonstrate how different schemes of information encoding are decoded with markedly different performance depending on the classification method applied. For the sake of simplicity, the data contained properties similar to those from a single slice acquisition of blood-oxygen-level-dependent (BOLD) fMRI. We crafted four datasets where embedded signals differentiated between an alternating sequences of time (referred to as states *A* and *B*), as is common to experimental tasks with a blocked design (Figure 2; MATLAB code for constructing and analyzing the datasets is available online at http://www.duke.edu/~pak5/). Each dataset consisted of a 12-by-12 matrix sampled at 240 time points in which consecutive blocks of 10 time points alternate between states *A* and *B*. Signal discriminating the two states was incorporated within a circle of radius 3 voxels above 10 dB white Gaussian noise. Finally, a spatial filter (Gaussian kernel with FWHM of 3 voxels) was applied to reflect the inherent smoothness of fMRI data.

In particular, we were interested in the relative sensitivity of each analytical approach to information with particular spatiotemporal properties. In the case of univariate analyses, we compared the mean of each voxel during state *A* and *B* using a two tailed paired *t*-test. Classification was performed using linear and radial basis function support vector machines implemented in libSVM (Chang and Lin, 2011). We implemented a searchlight approach (Kriegeskorte et al., 2006) constructing multiple classifiers using data from each sample and its four nearest neighbors. This approach highlights how spatial smoothing inherent in fMRI acquisition may impact detection of information of nearby sources. To produce a generalized estimate of accuracy, twelve-fold leave-one-out cross-validation was performed (i.e., the analyses were repeated twelve times, each time partitioning a different block of data for an independent test). The statistical significance of accuracy estimates were then computed using binomial tests against chance levels of performance. Statistical maps produced from univariate and MVPC analyses were then thresholded at a family wise error corrected level of *p* < 0.05. Additionally, to compare the relative sensitivity and specificity of different signal detection methods, the area under the receiver operating characteristic curve was computed for all analyses. The hit rate was calculated as the proportion of significant voxels above threshold within the source of information. The false positive rate was similarly calculated using the same procedure, only on data containing smooth noise – no informative signal was added.

In our first example (*univariate encoding*), we simulated a dataset whose spatial properties were well-matched to the strengths of univariate statistics. We focused on two key properties in constructing this simulation. First, spatial location is not relevant to the information contained in the signal, so independent random sampling from the circle should yield consistent results. Second, the voxels do not interact and can be considered one at a time without a loss of information. Thus, performing a statistical test on each sample separately should be sufficient to identify where the signal is contained. To meet these requirements, we made each sample within the circle deactivated during state *A* and activated state *B*, resulting in a square waveform. We found that all statistical approaches correctly identified the majority of voxels within the circle. The enhanced sensitivity of MVPC was evident around the boundary of the activated region, where spatial smoothing decreased the information content of voxels. While the univariate approach only identified voxels within the circle, the pattern classifiers could identify voxels just outside the boundary where only a small amount of information was present due to the smoothness of the data. Thus, for this simplest case, the advantage of MVPC was that of increased sensitivity compared to univariate techniques.

For our second exemplar dataset (*sparse encoding*), we simulated a sparsely distributed signal within an activated region by randomly retaining one quarter of the voxels within the circle and increasing their amplitude four fold, ensuring the total amount of signal within the source remained constant. In this case, only some spatial locations provide information that differentiates the two states. For this reason, univariate tests may fail to identify a sample with low or intermediate amplitude, whereas multivariate methods can utilize spatial information to successfully classify the data. Statistical analysis on this dataset revealed that while all three methods could successfully identify that task-related signal was present in the dataset, the univariate analysis failed to identify some regions within the circle.

In the third example (*inverted encoding*), our goal was to demonstrate a less conventional dependence on spatial location. To accomplish this, we made every sample contain signal but reversed its sign in half the voxels selected at random. By reversing the sign of activity in half of the voxels, both the sign of the signal and its spatial location are required to decode the current state. Consistent with the previous example, multivariate pattern classifiers excelled at decoding spatial information while univariate analysis failed to detect the majority of voxels within the source. Taken together, the results from examples two and three demonstrate the increased sensitivity of MVPC over univariate approaches, with linear and non-linear classifiers exhibiting similar performance.

For the fourth example (*interactive encoding*), we created a source in which information is carried exclusively through the interaction of voxels. This was accomplished by randomly distributing two signals throughout the circle in a fashion similar to example three, but allowing the direction of activation to vary over time. In state *A* the regions alternate between being positive or negative with opposite signs, whereas in state *B* neither region is active. Classifying the current state requires creating a model that incorporates the interactions between voxels, because the overall activation of any voxel (or the region as a whole) carries no information. As expected, statistical analysis revealed that non-linear classification was the only approach that successfully identified the source of information in the data.

## Expanding the Use of Pattern Classification

Consistent with its increasing prevalence in the literature, MVPC has several distinct advantages over conventional univariate approaches. It has greater sensitivity for identifying small effects, especially when the spatial distribution of activity is heterogeneous. This result is consistent with studies finding functional organization in brain structures, such as visual cortex (Kamitani and Tong, 2005) and the hippocampus (Hassabis et al., 2009), that had been previously missed using univariate methods. Studies using linear classifiers to extract unexpected effects have popularized pattern analysis. We found, however, that the benefits of MVPC go well beyond a simple increase in detection power. Linear classifiers provide access to spatial information on top of that carried in the mean level of activity, while non-linear classifiers reveal information likely to be carried in complex computational maps.

The defining properties of linear MVPC are its use of spatial information and focus on individual voxels. Because the distribution of neurons sampled in a voxel can vary with spatial location, spatially sparse fMRI activity is likely a result of heterogeneity in underlying neural populations. While univariate methods gloss over these distinctions by examining each voxel separately, linear classification carries intuitive advantages. As has been commonly noted (Kamitani and Tong, 2005; Norman et al., 2006; Pereira et al., 2009), linear classifiers pool the information contained within individual voxels. This is a useful property when the goal of classification is to leverage fine scale spatial organization in studying brain function because the location of voxels, rather than interactions between them, will drive performance of classification. The combination of these two properties allows the method to reveal information beyond univariate approaches (Jimura and Poldrack, 2012) in a manner that is straightforward to interpret. Thus, linear classification should be employed when neural signals do not interact and are expected to be in a fixed spatial configuration, as in the *sparse encoding* and *inverted encoding* simulations.

Non-linear classification, on the other hand, should be harnessed when cognitive states do not necessarily correspond to the activation of individual voxels, but instead have differential effects depending on the functional properties of those voxels. Because non-linear algorithms treat the activity of a voxel as part of a combinatorial code rather than a unitary piece of information, they are better served to decode more complex representations across association cortex (Hanson et al., 2004) that build upon basic features in primary sensory cortex (e.g., Kamitani and Tong, 2005).This capacity may prove critical in representing multiple different categories from more basic properties in a robust, efficient manner (Op De Beeck et al., 2008). Further, our results demonstrated that non-linear algorithms can identify combinatorial patterns that are not time invariant, but drastically change over time despite containing the same information content. These findings suggest that non-linear classification is an advantageous methodological choice when neural signals are highly intricate and vary over time, for example in *interactive encoding*.

Despite their benefits and unique capacities, both linear and non-linear classifiers utilize information contained in the activity and spatial location of a sample, which can lead them to show similar results in many cases. The performance of MVPC methods must be compared to univariate results before claims about spatial information can be made. More specifically, a linear classifier must reveal information beyond that which is detectable by univariate methods before the spatial location of inputs can be considered important. Similarly, non-linear classifiers do not only decode pattern activity; mean levels of activity or spatial location can also drive the performance of these learning algorithms. We found comparisons between analysis methods to be infrequent in our meta-analysis, with comparisons to univariate methods being made in 16 (13.9%) studies and comparisons between non-linear and linear algorithms in only 11 (9.6%) studies. This is especially important since sensitivity in MVPC varies with signal amplitude (Smith et al., 2011). Additionally, given that non-linear classifiers can properly model decision boundaries that linear classifiers are incapable of solving (e.g., the XOR problem or our related *interactive encoding* example), the application of non-linear classifiers may lead to refinement of models already established with linear approaches. Model comparisons are essential in revealing information contained in non-linear relationships above and beyond the capacity of linear classifiers. While linear classifiers can be successfully applied to improve the sensitivity of fMRI in cases of functionally organized neural activity, they need to be complemented with non-linear algorithms to be a true advance over traditional approaches.

## Conflict of Interest Statement

The authors declare that the research was conducted in the absence of any commercial or financial relationships that could be construed as a potential conflict of interest.
